# Hierarchical Fuzzy Adaptive Observer-Based Fault-Tolerant Consensus Tracking for High-Order Nonlinear Multi-Agent Systems Under Actuator and Sensor Faults

**DOI:** 10.3390/s26010252

**Published:** 2025-12-31

**Authors:** Lei Zhao, Shiming Chen

**Affiliations:** 1 School of Electrical and Automation Engineering, East China Jiao Tong University, Nanchang 330013, China; 105074@hsu.edu.cn; 2 School of Mechanical and Electrical Engineering, Huangshan University, Huangshan 245041, China

**Keywords:** nonlinear multi-agent systems, consensus tracking control, fault-tolerant control, fuzzy adaptive control, actuator and sensor faults

## Abstract

This paper investigates the consensus tracking problem for a class of high-order nonlinear multi-agent systems subject to actuator faults, sensor faults, unknown disturbances, and model uncertainties. To effectively address this problem, a hierarchical fault-tolerant control framework with fuzzy adaptive mechanisms is proposed. First, a distributed output predictor based on a finite-time differentiator is constructed for each follower to estimate the leader’s output trajectory and to prevent fault propagation across the network. Second, a novel state and actuator-fault observer is designed to reconstruct unmeasured states and detect actuator faults in real time. Third, a sensor-fault compensation strategy is integrated into a backstepping procedure, resulting in a fuzzy adaptive consensus-tracking controller. This controller guarantees the uniform boundedness of all closed-loop signals and ensures that the tracking error converges to a small neighborhood of the origin. Finally, numerical simulations validate the effectiveness and robustness of the proposed method in the presence of multiple simultaneous faults and disturbances.

## 1. Introduction

In recent years, distributed cooperative control of multi-agent systems (MASs) has drawn increasing research interest due to its broad range of engineering applications, including unmanned aerial vehicles, spacecraft attitude coordination, smart grids, and wireless sensor networks [1,2,3,4]. As one of the fundamental topics in this area, the consensus problem has been extensively explored. According to existing studies, consensus behaviors are generally categorized into leaderless consensus and leader-following consensus. In the leaderless case, all agents aim to reach a common agreement value through interactions with their neighbors, whereas in the leader-following case, followers are required to track the reference trajectory generated by a designated leader. Over the past decade, numerous control strategies have been proposed to address various forms of consensus for MASs [5,6,7,8,9].

Previous studies on consensus control have primarily focused on linear MASs [10,11,12]. However, many practical systems inherently possess nonlinear dynamics, such as single-link robotic manipulators [13] and unmanned aerial vehicles [14]. This makes the investigation of consensus control for nonlinear MASs more relevant and technically meaningful. In recent years, several control strategies have been developed to address nonlinear consensus problems [15,16,17,18]. For example, ref. [15] considered the $H_{\infty }$ consensus problem for nonlinear second-order MASs by employing inequality-based analysis. A finite-time distributed controller for nonlinear MASs satisfying the Lipschitz condition was reported in [16]. An event-triggered sliding-mode consensus scheme for second-order nonlinear MASs was developed in [17]. Ref. [18] addressed $H_{\infty }$ leader–following consensus of nonlinear MASs under arbitrary topologies using a T–S fuzzy modeling approach with guaranteed performance. Furthermore, when nonlinearities are accompanied by unknown dynamics or parametric uncertainties, the consensus problem becomes more challenging. Moreover, fuzzy logic systems (FLSs) and neural networks (NNs) have been widely used as universal approximators to cope with such uncertainties. For instance, ref. [19] employed FLSs to approximate unknown nonlinearities and studied consensus control for MASs with matched uncertainties. A finite-time adaptive fuzzy consensus control method for heterogeneous nonlinear MASs was proposed in [20] using FLSs and Lyapunov-based techniques. In [21], an adaptive NN-based backstepping framework was introduced to achieve consensus tracking for high-order nonlinear MASs.

In addition, the aforementioned studies did not take into account the presence of faults, including actuator or sensor failures. As MASs are increasingly deployed in various safety-critical fields, higher demands are being placed on their reliability and tracking accuracy. As is well known, MASs involve numerous actuators and sensors, intricate communication structures, and a higher likelihood of component malfunctions, which can lead to significant performance degradation or even severe system losses. Consequently, developing effective fault-tolerant control (FTC) strategies for MASs is of both theoretical importance and practical engineering value, and remains a pressing research topic in the MASs community. To enhance system stability and reliability, numerous effective FTC methods have been developed for MASs [22,23,24,25,26,27,28,29]. To address parameter uncertainties and transient instability induced by hybrid actuator faults, ref. [30] proposed a novel adaptive FTC approach that integrates backstepping with dynamic surface control to compensate for unknown nonlinear actuator faults. In [31], an adaptive fixed-time FTC scheme was presented for nonlinear MASs, in which a Nussbaum-based approach is employed to construct an effective actuator fault compensation mechanism. For nonstrict-feedback nonlinear MASs subject to intermittent actuator faults, an adaptive fuzzy consensus FTC method was proposed in [32]. An adaptive NNs-based event-triggered control scheme was proposed for nonlinear MASs in [33], where input saturation, disturbances, and sensor faults were considered simultaneously. It is worth noting that most existing FTC methods address either actuator faults or sensor faults independently. In practice, however, actuator and sensor faults often occur simultaneously, making the problem significantly more complex. Although the resilient leader–follower tracking problem under simultaneous sensor and actuator faults has been investigated by using adaptive control approaches in [34,35], these works do not consider high-order nonlinear MASs. This practical challenge motivates the study presented in this paper.

In this paper, we address the distributed fault-tolerant consensus tracking problem for high-order nonlinear MASs subject to unknown time-varying actuator and sensor faults and external disturbances. Following a hierarchical design philosophy, a predictor/observer-based fuzzy adaptive FTC scheme is developed by integrating fuzzy logic systems, adaptive estimation, and backstepping control. In comparison with existing FTC methods for MASs, the main contributions of this work are summarized as follows:

1. The proposed hierarchical FTC framework achieves a clean separation between predictor/observer design and controller synthesis, enabling each agent to accomplish distributed consensus tracking without modifying its original controller structure. This decoupled architecture not only simplifies the cooperative control design under complex nonlinear dynamics and multiple fault types, but also enhances scalability, flexibility, and practical applicability in large-scale MASs.
2. Compared with the methods in [30,31,32,33], which handle either actuator faults or sensor faults independently, these approaches become difficult to apply when both types of faults occur simultaneously, often resulting in degraded performance or even instability. Although [34,35] consider concurrent actuator and sensor failures for MASs, extending such FTC schemes to high-order nonlinear MASs is nontrivial. Motivated by these limitations, this paper develops an adaptive fuzzy observer-based FTC scheme for MASs that can effectively cope with simultaneous actuator and sensor faults while preserving the desired consensus tracking performance.
3. Unlike methods that rely on explicit fault-detection and isolation modules [22,25], the proposed approach embeds adaptive estimation directly into the predictor–observer– controller loop. This enables real-time reconstruction and compensation of actuator and sensor faults, thereby reducing system complexity and improving practicality in multi-fault nonlinear MAS environments.

The remainder of this article is organized as follows: Section 2 presents the necessary preliminaries and formulates the problem. Section 3 develops the proposed control framework and provides the main theoretical results. Section 4 illustrates the effectiveness of the method through numerical simulations. Section 5 concludes this paper.

Notations: $R^{n}$ and $R^{n\times m}$ denote the sets of *n*-dimensional real vectors and real matrices of size n×m, respectively. |·| represents the absolute value, and $∥\cdot ∥$ the Euclidean or induced norm. $\lambda_{\min}\left(X\right)$ and $\lambda_{\max}\left(X\right)$ denote the smallest and largest eigenvalues of a square matrix *X*. $\sigma_{\min}\left(\cdot \right)$ stands for the minimum singular value. $diag\left\{x_{1},\cdots ,x_{n}\right\}$ is a diagonal matrix with diagonal entries $x_{1},\cdots ,x_{n}$. For any matrix *X*, *X^T^* denotes its transpose.

## 2. Preliminary and Problem Statement

### 2.1. Graph Theory

Let G=(V,E,A) be a directed graph, where $V=\left\{v_{1},\ldots ,v_{N}\right\}$ denotes the node set, $E=\left\{\left(v_{i},v_{j}\right),|,v_{i},v_{j}\in V\right\}$ represents the edge set, and $A={\left[a_{ij}\right]}_{N\times N}$ is the corresponding adjacency matrix. An edge $(v_{j},v_{i})\in E$ exists and $a_{ij}=1$ if node $v_{i}$ receives information from node $v_{j}$; otherwise, $a_{ij}=0$. The neighbor set of node $v_{i}$ is defined as $N_{i}=\left\{v_{j},|,\left(v_{j},v_{i}\right)\in E\right\}$. The Laplacian matrix of the digraph G is denoted by $L={\left[l_{ij}\right]}_{N\times N}$, where $l_{ij}=-a_{ij}$ for i≠j, and $l_{ii}=\sum_{j\in Ni}a_{ij}$. A directed spanning tree exists in G if there is at least one root node that has a directed path to every other node.

For the leader–follower communication topology, $\bar{G}$ denotes the augmented graph that includes a leader node $v_{0}$ and the follower set V. The diagonal matrix $B=\mathrm{diag}\left\{b_{1},\ldots ,b_{N}\right\}$ is introduced to indicate the communication links from the leader, where $b_{i}=1$ means that follower $v_{i}$ can directly access the leader’s information, and $b_{i}=0$ otherwise.

### 2.2. Problem Statement

Consider a class of high-order nonlinear MASs composed of one leader and *N* followers. The dynamics of the *i*-th follower are described by

(1) $$\left\{\begin{array}{c} {\dot{\varsigma }}_{i,m}=\varsigma_{i,m+1}+\psi_{i,m}\left({\bar{\varsigma }}_{i,m}\right)+d_{i,m}\\ {\dot{\varsigma }}_{i,n_{i}}=u_{i}+\psi_{i,n_{i}}\left({\bar{\varsigma }}_{i,n_{i}}\right)+d_{i,n_{i}}\\ y_{i}=\varsigma_{i,1},m=1,\ldots ,n_{i}-1,i=1,\ldots ,N \end{array}\right.$$

where ${\bar{\varsigma }}_{i,m}={\left[\varsigma_{i,1},\varsigma_{i,2},\cdots ,\varsigma_{i,m}\right]}^{T}\in R^{m}$, $u_{i}$ and $y_{i}$ denote the control input and system output, respectively. $\psi_{i,m}\left({\bar{\varsigma }}_{i,m}\right):R^{m}\to R$ represents unknown smooth nonlinearities. The external disturbances $d_{i,m}$ satisfies $\left|d_{i,m}\right|\leq d_{i,m}^{*}$, where $d_{i,m}^{*}$ is an unknown positive constant. The leader provides a reference output trajectory denoted by $y_{d}\in R$.

Here, we assume that the *i*-th follower is simultaneously subjected to unknown, time-varying actuator and sensor faults. The fault models are described as follows:

(2) $$\left\{\begin{array}{c} y_{i}^{f}=\beta_{si}\left(t\right)y_{i}+f_{si}\left(t\right)\\ u_{i}^{f}=\beta_{ai}\left(t\right)u_{i}+f_{ai}\left(t\right) \end{array}\right.$$

where $y_{i}^{f}$ is the faulty output measurement and $0<\beta_{si}\left(t\right)\leq 1$ denotes the sensor sensitivity. $u_{i}^{f}$ is the actuator-fault output and $0<\beta_{ai}\left(t\right)\leq 1$ represents the loss-of-effectiveness factor. $f_{si}\left(t\right)$ and $f_{ai}\left(t\right)$ denote additive bias faults in the sensor and actuator, respectively.

Motivated by [36], the actuator and sensor faults in (2) can be equivalently expressed as follows:

(3) $$\left\{\begin{array}{c} y_{i}^{f}=y_{i}+\delta_{si}\\ u_{i}^{f}=u_{i}+\delta_{ai} \end{array}\right.$$

where $\delta_{si}=\left(\beta_{si}\left(t\right)-1\right)y_{i}+f_{si}\left(t\right)$, $\delta_{ai}=\left(\beta_{ai}\left(t\right)-1\right)u_{i}+f_{ai}\left(t\right)$ represent the lumped sensor-fault and actuator-fault, respectively.

Substituting (3) into (1), the system described by (1) can be rewritten as follows:

(4) $$\left\{\begin{array}{c} {\dot{\varsigma }}_{i,m}=\varsigma_{i,m+1}+\psi_{i,m}\left({\bar{\varsigma }}_{i,m}\right)+d_{i,m}\\ {\dot{\varsigma }}_{i,n_{i}}=u_{i}+\delta_{ai}+\psi_{i,n_{i}}\left({\bar{\varsigma }}_{i,n_{i}}\right)+d_{i,n_{i}}\\ y_{i}^{f}=\varsigma_{i,1}+\delta_{si} \end{array}\right.$$

Objective: This work develops a hierarchical FTC scheme capable of handling unmodeled dynamics, actuator faults, and sensor faults while ensuring that each follower tracks the leader’s trajectory $y_{d}$, regardless of the disturbances. Specifically, there exists $\varepsilon_{y}>0$ such that $\left|y_{i}-y_{d}\right|\leq \varepsilon_{y}$.

To facilitate the controller design, the following assumptions and lemmas are introduced.

**Assumption** **1.** *The augmented graph $\bar{G}$ contains a directed spanning tree with the leader node as the root.*

**Assumption** **2.** *The leader’s trajectory $y_{d}$ is piecewise continuous, and $y_{d}$, ${\dot{y}}_{d}$, and ${\ddot{y}}_{d}$ are bounded.*

**Assumption** **3.** *For each follower, the function $\psi_{i,m}\left(\cdot \right)$ satisfies the Lipschitz condition. That is, there exists positive constant $\gamma_{i,m}>0$ such that $\left|\psi_{i,m}\left(X\right)-\psi_{i,m}\left(Y\right)\right|\leq \gamma_{i,m}∥X-Y∥$, for all X, Y in the domain of interest.*

**Assumption** **4.** *the time-varying fault functions $\delta_{si}$ and $\delta_{ai}$ and their derivatives are bounded, There exist positive constants ${\bar{\delta }}_{si}$, ${\bar{\bar{\delta }}}_{si}$, ${\bar{\delta }}_{ai}$, and ${\bar{\bar{\delta }}}_{ai}$, such that $\left|\delta_{si}\right|\leq {\bar{\delta }}_{si}$, $\left|{\dot{\delta }}_{si}\right|\leq {\bar{\bar{\delta }}}_{si}$, $\left|\delta_{ai}\right|\leq {\bar{\delta }}_{ai}$, and $\left|{\dot{\delta }}_{ai}\right|\leq {\bar{\bar{\delta }}}_{ai}$.*

**Remark** **1.** *These assumptions are commonly adopted in existing studies. Assumption 1 is a standard condition widely employed to guarantee leader–follower consensus tracking of MASs in much of the literature [19,21]. Assumptions 2 and 3 have also been utilized in previous works [37,38,39].*

**Remark** **2.** *Assumptions 4 is commonly adopted in fault-tolerant control of engineering systems and are practically reasonable, such as [34,35]. In real applications, actuator and sensor faults such as loss of effectiveness, bias faults, or degradation processes are constrained by physical limitations, protection mechanisms, and safety regulations. Moreover, abrupt changes in fault signals are typically filtered by system dynamics, hardware bandwidth limitations, or fault accommodation mechanisms, which makes the fault signals and their rates of change bounded. These assumptions ensure implementability of the proposed adaptive observer and controller while maintaining robustness against realistic fault behaviors.*

**Lemma** **1** ([40]). *If the augmented graph $\bar{G}$ contains a directed spanning tree, the matrix L+B is invertible, and all its eigenvalues have positive real part.*

**Lemma** **2** ([41]). *Let ϕ(ς) be a continuous function defined on a compact set *Ω*. For any ε>0, there exists a FLS such that*

(5) $$\underset{\varsigma \in \Omega }{\sup}\left|\psi \left(\varsigma \right)-\theta^{T}\phi \left(\varsigma \right)\right|\leq \varepsilon$$

*where $\theta^{T}=\left[\theta_{1},\ldots ,\theta_{N}\right]$ is the parameter vector. $\phi^{T}\left(\varsigma \right)=\left[\phi_{1}\left(\varsigma \right),\ldots ,\phi_{N}\left(\varsigma \right)\right]/\sum_{i=1}^{N}\phi_{i}\left(\varsigma \right)$ is the fuzzy basis function vector, and $\phi_{i}\left(\varsigma \right)$ are defined as*

$$\phi_{i}\left(\varsigma \right)=\exp\left[\frac{-{\left(\varsigma -\pi_{i}\right)}^{T}\left(\varsigma -\pi_{i}\right)}{\iota_{i}^{2}}\right],$$

*where $\iota_{i}$ is the width of the Gaussian function, and $\pi_{i}={\left[\pi_{i,1},\ldots ,\pi_{i,l}\right]}^{T}$ is the center vector.*

## 3. Main Results

### 3.1. Differentiator-Based Distributed Output Predictor Design

In this subsection, a differentiator-based distributed output predictor is designed for each follower by utilizing local neighborhood information. The distributed predictor for the *i*-th follower is designed as follows:

(6) $$\left\{\begin{array}{c} {\dot{\xi }}_{i,1}=\xi_{i,2}\\ {\dot{\xi }}_{i,2}=-\iota^{2}\mathrm{sgn}\left(\xi_{i,1}-y_{N_{i}}\right){\left|\xi_{i,1}-y_{N_{i}}\right|}^{\beta }-\iota \xi_{i,2}\\ {\dot{\bar{y}}}_{i}=\frac{1}{\rho_{i}}\left(\xi_{i,2}-k_{0}\sum_{j=1}^{N}a_{ij}\left({\bar{y}}_{i}-{\bar{y}}_{j}\right)-k_{0}b_{i}\left({\bar{y}}_{i}-y_{d}\right)\right) \end{array}\right.$$

where $\xi_{i,1}$ and $\xi_{i,2}$ represent the states of the tracking differentiator (TD), and ${\bar{y}}_{i}$ denotes the predictor state. The auxiliary signal $y_{N_{i}}=\sum_{j=1}^{N}a_{ij}{\bar{y}}_{j}+b_{i}y_{d}$ is generated using the available neighborhood information. Here, $k_{0}>0$, ι>0, and 0<β<1 are design constants, and $\rho_{i}=\sum_{j=1}^{N}a_{ij}+b_{i}$.

**Remark** **3.** *The tracking differentiator in (6) is designed based on the finite-time differentiator proposed in [42]. It guarantees that the estimation error $\xi_{i,2}-{\dot{y}}_{N_{i}}$ converges to a bounded neighborhood of the origin within finite time. The ultimate bound depends on the differentiator parameters (ι,β) and the boundedness of the derivatives of $y_{N_{i}}$. For the subsequent predictor and observer design, only the boundedness of the differentiator error is required, and an explicit closed-form expression of the bound is not necessary.*

**Theorem** **1.** *Suppose that Assumption 1 holds. Then, for the distributed output predictor given in (6), the predictor state ${\bar{y}}_{i}$ can track the leader’s trajectory $y_{d}$ with a sufficiently small tracking error.*

**Proof.**  Define the consensus error of follower *i* as follows:

(7) $$e_{i}=\sum_{j=1}^{N}a_{ij}\left({\bar{y}}_{i}-{\bar{y}}_{j}\right)+b_{i}\left({\bar{y}}_{i}-y_{d}\right),i=1,2,\ldots ,N.$$

Differentiating (7) and using the predictor dynamics yields

(8) $${\dot{e}}_{i}=\xi_{i,2}-k_{0}e_{i}-{\dot{y}}_{N_{i}}$$

Solving (8) gives

(9) $$e_{i}=e^{-k_{0}t}e_{i}\left(0\right)+\int_{0}^{t}e^{-k_{0}\left(t-\tau \right)}\left(\xi_{i,2}-{\dot{y}}_{N_{i}}\right)d\tau$$

where $e_{i}\left(0\right)$ is the initial conditions of the consensus error.

From the finite-time convergence property of the tracking differentiator [42], there exists a constant $o_{i}>0$ such that $\lim_{t\to \infty }\left|\xi_{i,2}-{\dot{y}}_{N_{i}}\right|\leq o_{i}$.

From (9), we obtain

(10) $$\lim_{t\to \infty }\left|e_{i}\right|\leq o_{i}/k_{0}.$$

Let $e^{T}=\left[e_{1},e_{2},\ldots ,e_{N}\right]$, ${\bar{y}}^{T}=\left[{\bar{y}}_{1},{\bar{y}}_{2},\ldots ,{\bar{y}}_{N}\right]$. Based on Lemma 1, it follows that

(11) $$∥\bar{y}-1_{N}y_{d}∥\leq ∥e∥/\sigma_{\min}\left(L+B\right)$$

where $1_{N}$ denotes the *N*-dimensional vector of all ones, and $\sigma_{\min}\left(L+B\right)$ represents the smallest singular value of the matrix L+B.

From (10) and (11), it follows that by selecting a sufficiently large coupling gain $k_{0}$ and suitable differentiator parameters (ι,β), the predictor state ${\bar{y}}_{i}$ can track $y_{d}$ with an arbitrarily small tracking error. □

### 3.2. States and Actuator-Fault Observer Design

Let ${\hat{\bar{\varsigma }}}_{i,m}={\left[{\hat{\varsigma }}_{i,1},{\hat{\varsigma }}_{i,2},\ldots ,{\hat{\varsigma }}_{i,m}\right]}^{T}$ denote the estimate of ${\bar{\varsigma }}_{i,m}={\left[\varsigma_{i,1},\varsigma_{i,2},\ldots ,\varsigma_{i,m}\right]}^{T}$. The system (4) can be rewritten as follows:

(12) $$\left\{\begin{array}{c} {\dot{\varsigma }}_{i,m}=\varsigma_{i,m+1}+\psi_{i,m}\left({\hat{\bar{\varsigma }}}_{i,m}\right)+\Delta \psi_{i,m}+d_{i,m}\\ {\dot{\varsigma }}_{i,n_{i}}=u_{i}+\delta_{ai}+\psi_{i,n_{i}}\left({\hat{\bar{\varsigma }}}_{i,n_{i}}\right)+\Delta \psi_{i,n_{i}}+d_{i,n_{i}}\\ y_{i}^{f}=y_{i}+\delta_{si} \end{array}\right.$$

where $\Delta \psi_{i,m}=\psi_{i,m}\left({\bar{\varsigma }}_{i,m}\right)-\psi_{i,m}\left({\hat{\bar{\varsigma }}}_{i,m}\right)$, $m=1,\ldots ,n_{i}$.

According to Lemma 2, the unknown function $\psi_{i,m}\left({\hat{\bar{\varsigma }}}_{i,m}\right)$ can be approximated by FLS as follows:

(13) $${\hat{\psi }}_{i,m}\left(\left.{\hat{\bar{\varsigma }}}_{i,m}\right|\theta_{i,m}\right)=\theta_{i,m}^{T}\phi_{i,m}\left({\hat{\bar{\varsigma }}}_{i,m}\right)$$

where $\theta_{i,m}$ is the adjustable parameter vector and $\phi_{i,m}\left(\cdot \right)$ is the corresponding fuzzy basis function vector.

The optimal parameter vector $\theta_{i,m}^{*}$ is defined by

(14) $$\theta_{i,m}^{*}=\arg\;\min_{\theta_{i,m}\in \Omega_{i,m}}\left[\underset{{\hat{\bar{\varsigma }}}_{i,m}\in U_{i,m}}{\sup}\left|{\hat{\psi }}_{i,m}\left(\left.{\hat{\bar{\varsigma }}}_{i,m}\right|\theta_{i,m}\right)-\psi_{i,m}\left({\hat{\bar{\varsigma }}}_{i,m}\right)\right|\right]$$

where $\Omega_{i,m}$ and $U_{i,m}$ are the compact regions for $\theta_{i,m}$ and ${\hat{\bar{\varsigma }}}_{i,m}$, respectively. The corresponding minimum approximation error is

(15) $$\varepsilon_{i,m}=\psi_{i,m}\left({\hat{\bar{\varsigma }}}_{i,m}\right)-{\hat{\psi }}_{i,m}\left(\left.{\hat{\bar{\varsigma }}}_{i,m}\right|\theta_{i,m}^{*}\right),$$

**Assumption** **5.** There exist positive constants $\varepsilon_{i,m}^{*}>0$ such that $\left|\varepsilon_{i,m}\right|\leq \varepsilon_{i,m}^{*}$, for all 1≤i≤N, $1\leq m\leq n_{i}$.

Substituting (13) and (15) into (12) yields

(16) $$\left\{\begin{array}{c} {\dot{\varsigma }}_{i,m}=\varsigma_{i,m+1}+{\hat{\psi }}_{i,m}\left(\left.{\hat{\bar{\varsigma }}}_{i,m}\right|\theta_{i,m}^{*}\right)+\varepsilon_{i,m}+\Delta \psi_{i,m}+d_{i,m}\\ {\dot{\varsigma }}_{i,n_{i}}=u_{i}+\delta_{ai}+{\hat{\psi }}_{i,m}\left(\left.{\hat{\bar{\varsigma }}}_{i,n_{i}}\right|\theta_{i,n_{i}}^{*}\right)+\varepsilon_{i,n_{i}}+\Delta \psi_{i,n_{i}}+d_{i,n_{i}}\\ y_{i}^{f}=y_{i}+\delta_{si} \end{array}\right.$$

To address unmeasured system states and actuator faults, based on (16), an observer for estimating both system states and actuator faults is constructed as follows:

(17) $$\left\{\begin{array}{c} {\dot{\hat{\varsigma }}}_{i,m}={\hat{\varsigma }}_{i,m+1}+{\hat{\psi }}_{i,m}\left(\left.{\hat{\bar{\varsigma }}}_{i,m}\right|\theta_{i.m}\right)+k_{m}{\hat{l}}^{m}\left(y_{i}^{f}-{\hat{\delta }}_{si}-{\hat{y}}_{i}\right)\\ {\dot{\hat{\varsigma }}}_{i,n_{i}}=u_{i}+{\hat{\delta }}_{ai}+{\hat{\psi }}_{i,m}\left(\left.{\hat{\bar{\varsigma }}}_{i,n_{i}}\right|\theta_{i,n_{i}}\right)+k_{n_{i}}{\hat{l}}^{n_{i}}\left(y_{i}^{f}-{\hat{\delta }}_{si}-{\hat{y}}_{i}\right)\\ {\dot{\hat{\delta }}}_{ai}=-\sigma_{ai}\upsilon_{ai}{\hat{\delta }}_{ai}+k_{ai}{\hat{l}}^{-1}\left(y_{i}^{f}-{\hat{\delta }}_{si}-{\hat{y}}_{i}\right)\\ {\hat{y}}_{i}={\hat{\varsigma }}_{i,1}w \end{array}\right.$$

where $k_{m}>0$, $\hat{l}>0$, $\sigma_{ai}>0$, $\upsilon_{ai}>0$, and $k_{ai}>0$ are design parameters. The signal ${\hat{\delta }}_{ai}$ denotes the estimate of $\delta_{ai}$, and ${\hat{\delta }}_{si}$ is the estimate of $\delta_{si}$.

Let ${\tilde{\varsigma }}_{i,m}=\left(\varsigma_{i,m}-{\hat{\varsigma }}_{i,m}\right)/{\hat{l}}^{m}$, we can obtain

(18) $${\dot{\tilde{\varsigma }}}_{i}=\hat{l}A_{i}{\tilde{\varsigma }}_{i}+{\hat{L}}_{i}\Theta_{i}+{\hat{L}}_{i}\varepsilon_{i}+{\hat{L}}_{i}\Delta \psi_{i}-K_{i}{\tilde{\delta }}_{si}+E_{i}{\tilde{\delta }}_{ai}+{\hat{L}}_{i}d_{i}$$

where ${\tilde{\varsigma }}_{i}^{T}=\left[{\tilde{\varsigma }}_{i,1},{\tilde{\varsigma }}_{i,2},\ldots ,{\tilde{\varsigma }}_{i,n_{i}}\right]$, ${\hat{L}}_{i}=diag\left\{\left(1/\hat{l}\right),\left(1/{\hat{l}}^{2}\right),\ldots ,\left(1/{\hat{l}}^{n_{i}}\right)\right\}$, $K_{i}={\left[k_{1},k_{2}\ldots ,k_{n_{i}}\right]}^{T}$, $E_{i}={\left[0,\ldots ,0,(1/{\hat{l}}^{n_{i}})\right]}^{T}\in R^{n_{i}}$, $\Delta \psi_{i}={\left[\Delta \psi_{i,1},\Delta \psi_{i,2},\ldots ,\Delta \psi_{i,n_{i}}\right]}^{T}$, $\varepsilon_{i}={\left[\varepsilon_{i,1},\varepsilon_{i,2}\ldots ,\varepsilon_{i,n_{i}}\right]}^{T}$, $d_{i}={\left[d_{i,1},d_{i,2}\ldots ,d_{i,n_{i}}\right]}^{T}$, $\Theta_{i}={\left[{\tilde{\theta }}_{i,1}^{T}\phi_{i,1}\left({\hat{\bar{\varsigma }}}_{i,1}\right),{\tilde{\theta }}_{i,2}^{T}\phi_{i,2}\left({\hat{\bar{\varsigma }}}_{i,2}\right),\ldots ,{\tilde{\theta }}_{i,n_{i}}^{T}\phi_{i,n_{i}}\left({\hat{\bar{\varsigma }}}_{i,n_{i}}\right)\right]}^{T}$, ${\tilde{\theta }}_{i,m}=\theta_{i,m}^{*}-\theta_{i,m}$, ${\tilde{\delta }}_{ai}=\delta_{ai}-{\hat{\delta }}_{ai}$, ${\tilde{\delta }}_{si}=\delta_{si}-{\hat{\delta }}_{si}$, and $A_{i}=\left[\begin{array}{cccc} -k_{1} & & & \\ \vdots & & I_{n_{i}-1} & \\ -k_{n_{i}} & 0 & \ldots & 0 \end{array}\right]$.

Choose $K_{i}$ and $\hat{l}$ such that $A_{i}$ is Hurwitz. Then, for any given positive definite matrix $Q_{i}$, there exists a positive definite matrix $P_{i}=P_{i}^{T}$ satisfying

(19) $$A_{i}^{T}P_{i}+P_{i}A_{i}=-Q_{i}.$$

Consider the Lyapunov function candidate

(20) $$V_{i,0}={\tilde{\varsigma }}_{i}^{T}P_{i}{\tilde{\varsigma }}_{i}+\frac{1}{2\upsilon_{ai}}{\tilde{\delta }}_{ai}^{2}.$$

Taking the time derivative of $V_{i,0}$ along (18) and using (19) yields

(21) $$\begin{array}{cc} {\dot{V}}_{i,0} & ={\tilde{\varsigma }}_{i}^{T}\left(\hat{l}A_{i}^{T}P_{i}+P_{i}A_{i}\hat{l}\right){\tilde{\varsigma }}_{i}+2{\tilde{\varsigma }}_{i}^{T}P_{i}\left(\hat{L}\Theta_{i}+{\hat{L}}_{i}\varepsilon_{i}+{\hat{L}}_{i}\Delta \psi_{i}-K_{i}{\tilde{\delta }}_{si}+E_{i}{\tilde{\delta }}_{ai}+{\hat{L}}_{i}d_{i}\right)\\ \; & \;+\frac{1}{\upsilon_{ai}}{\tilde{\delta }}_{ai}{\dot{\delta }}_{ai}-\frac{1}{\upsilon_{ai}}{\tilde{\delta }}_{ai}\left(-\sigma_{ai}\upsilon_{ai}{\hat{\delta }}_{ai}+k_{ai}{\hat{l}}^{-1}\left(y_{i}^{f}-{\hat{\delta }}_{si}-{\hat{y}}_{i}\right)\right) \end{array}$$

On the basis of Assumption 3, Young’s inequality and the fact that $0\leq \phi_{i,m}^{T}\phi_{i,m}\leq 1$, we can obtain

(22) $$\begin{array}{cc} 2{\tilde{\varsigma }}_{i}^{T}P_{i}{\hat{L}}_{i}\left(\varepsilon_{i}+d_{i}\right)\leq & \;2{∥{\tilde{\varsigma }}_{i}∥}^{2}+{∥P_{i}∥}^{2}{∥{\hat{L}}_{i}∥}^{2}\left({∥\varepsilon_{i}^{*}∥}^{2}+{∥d_{i}^{*}∥}^{2}\right),\;\\ 2{\tilde{\varsigma }}_{i}^{T}P_{i}{\hat{L}}_{i}\Delta \psi_{i}\leq & \;{∥{\tilde{\varsigma }}_{i}∥}^{2}+{∥P_{i}∥}^{2}{∥{\hat{L}}_{i}∥}^{2}\sum_{m=1}^{n_{i}}\gamma_{i,m}^{2}{∥{\tilde{\varsigma }}_{i}∥}^{2},\;\\ 2{\tilde{\varsigma }}_{i}^{T}P_{i}K_{i}{\tilde{\delta }}_{si}\leq & \;{∥{\tilde{\varsigma }}_{i}∥}^{2}+{∥P_{i}K_{i}∥}^{2}{\tilde{\delta }}_{si}^{2},\;\\ 2{\tilde{\varsigma }}_{i}^{T}P_{i}E_{i}{\tilde{\delta }}_{ai}\leq & \;{∥{\tilde{\varsigma }}_{i}∥}^{2}+{∥P_{i}E_{i}∥}^{2}{\tilde{\delta }}_{ai}^{2},\;\\ {\tilde{\delta }}_{ai}{\dot{\delta }}_{ai}\leq & \;\frac{1}{2}{\tilde{\delta }}_{ai}^{2}+\frac{1}{2}{\bar{\bar{\delta }}}_{ai}^{2},\;\\ {\tilde{\delta }}_{ai}{\hat{\delta }}_{ai}\leq & -\frac{1}{2}{\tilde{\delta }}_{ai}^{2}+\frac{1}{2}{\bar{\delta }}_{ai}^{2},\;\\ {\hat{l}}^{-1}{\tilde{\delta }}_{ai}\left(y_{i}^{f}-{\hat{\delta }}_{si}-{\hat{y}}_{i}\right)\leq & \;{\tilde{\delta }}_{ai}^{2}+\frac{1}{2}{∥{\tilde{\varsigma }}_{i}∥}^{2}+\frac{1}{2{\hat{l}}^{2}}{\tilde{\delta }}_{si}^{2},\;\\ 2{\tilde{\varsigma }}_{i}^{T}P_{i}{\hat{L}}_{i}\Theta_{i}\leq & \;n_{i}{∥{\tilde{\varsigma }}_{i}∥}^{2}+{∥P_{i}∥}^{2}{∥{\hat{L}}_{i}∥}^{2}\sum_{m=1}^{n_{i}}{\tilde{\theta }}_{i,m}^{T}{\tilde{\theta }}_{i,m}. \end{array}$$

Substituting (22) into (21) yields

(23) $${\dot{V}}_{i,0}\leq -c_{i}{∥{\tilde{\varsigma }}_{i}∥}^{2}-c_{ai}{\tilde{\delta }}_{ai}^{2}+c_{si}{\tilde{\delta }}_{si}^{2}+{∥P_{i}∥}^{2}{∥{\hat{L}}_{i}∥}^{2}\sum_{m=1}^{n_{i}}{\tilde{\theta }}_{i,m}^{T}{\tilde{\theta }}_{i,m}+D_{i,0},$$

where $c_{i}=\hat{l}\lambda_{\min}\left(Q_{i}\right)-5-n_{i}-{∥P_{i}∥}^{2}{∥{\hat{L}}_{i}∥}^{2}\sum_{m=1}^{n}\gamma_{i,m}^{2}-\frac{k_{ai}}{2\upsilon_{ai}}$, $c_{ai}=\frac{1}{2}\sigma_{ai}-\frac{1}{2\upsilon_{ai}}-\frac{k_{ai}}{\upsilon_{ai}}-{∥P_{i}E_{i}∥}^{2}$, $c_{si}={∥P_{i}K_{i}∥}^{2}+\frac{k_{ai}}{2\upsilon_{ai}l^{2}}$, and $D_{i,0}={∥P_{i}∥}^{2}{∥{\hat{L}}_{i}∥}^{2}\left({∥\varepsilon_{i}^{*}∥}^{2}+{∥d_{i}^{*}∥}^{2}\right)+\frac{1}{2\upsilon_{ai}}{\bar{\bar{\delta }}}_{ai}^{2}+\frac{1}{2}\sigma_{ai}{\bar{\delta }}_{ai}^{2}$.

**Remark** **4.** *Unlike [37,38], the true system output $y_{i}$ is not directly measurable in the presence of sensor faults. However, $y_{i}^{f}-{\hat{\delta }}_{si}$ is available after estimating the sensor-fault term. The observer constructed in this work utilizes the faulty measurement $y_{i}^{f}$, the estimated sensor fault ${\hat{\delta }}_{si}$, and the predictor output ${\bar{y}}_{i}$ to simultaneously estimate the system states and actuator fault.*

**Remark** **5.** *The observer gains in (17) can be selected following standard high-gain observer and adaptive control design principles. Specifically, the gains $k_{m}$ and the scaling parameter $\hat{l}$ are chosen such that the matrix $A_{i}$ is Hurwitz, which guarantees exponential convergence of the nominal observer error dynamics. Larger values of $\hat{l}$ and $k_{m}$ generally improve the convergence speed of the state estimation, but excessively large gains may amplify measurement noise. The parameters $\sigma_{ai}$ and $\upsilon_{ai}$ are selected to ensure sufficient damping and boundedness of the actuator fault estimation error, while $k_{ai}$ determines the convergence rate of the actuator fault estimate. In practice, these gains can be tuned iteratively to balance estimation performance and robustness, as demonstrated in the simulation studies.*

### 3.3. Adaptive Fault-Tolerant Control Consensus Protocol Design and Stability Analysis

In this subsection, an adaptive fault-tolerant consensus control protocol is developed by integrating the previously designed distributed output predictor, state observer, and actuator fault observer through the backstepping technique. To facilitate the recursive design process, a coordinate transformation is introduced as follows:

(24) $$\begin{array}{cc} z_{i,1}=y_{i}-{\bar{y}}_{i}= & \;y_{i}^{f}-{\bar{y}}_{i}-{\hat{\delta }}_{si}-{\tilde{\delta }}_{si}={\tilde{z}}_{i,1}-{\tilde{\delta }}_{si}\\ z_{i,m}= & \;{\hat{\varsigma }}_{i,m}-\zeta_{i,m}\\ \chi_{i,m}= & \;\zeta_{i,m}-\alpha_{i,m-1},i=1,2,\cdots ,N,m=2,\cdots ,n_{i} \end{array}$$

where ${\tilde{z}}_{i,1}=y_{i}^{f}-{\bar{y}}_{i}-{\hat{\delta }}_{si}$ is the measurable part of the tracking error, $\alpha_{i,m-1}$ denotes the virtual controller associated with step m−1, and $\zeta_{i,m}$ together with $\chi_{i,m}$ are the outputs and errors of the first-order filters introduced below.

To avoid the “explosion of complexity” typically encountered in backstepping designs, a first-order filter is employed for each virtual control signal. The dynamics of the filter are given by

(25) $$\tau_{i,m}{\dot{\zeta }}_{i,m}+\zeta_{i,m}=\alpha_{i,m-1},\zeta_{i,m}\left(0\right)=\alpha_{i,m-1}\left(0\right)$$

where $\tau_{i,m}$ is a given constant.

The controller is derived as the last step of the backstepping process.

Step 1: From (24), we have

(26) $$\begin{array}{cc} {\dot{z}}_{i,1}= & \;\varsigma_{i,2}+\psi_{i,1}\left({\bar{\varsigma }}_{i,1}\right)+d_{i,1}-{\dot{\bar{y}}}_{i}\\ = & \;z_{i,2}+\chi_{i,2}+\alpha_{i,1}+{\hat{l}}^{2}{\tilde{\varsigma }}_{i,2}+\theta_{i,1}^{T}\phi_{i,1}\left({\hat{\bar{\varsigma }}}_{i,1}\right)+{\tilde{\theta }}_{i,1}^{T}\phi_{i,1}\left({\hat{\bar{\varsigma }}}_{i,1}\right)+\varepsilon_{i,1}+\Delta \psi_{i,1}+d_{i,1}-{\dot{\bar{y}}}_{i} \end{array}$$

Choose the Lyapunov function

(27) $$V_{i,1}=V_{i,0}+\frac{1}{2}z_{i,1}^{2}+\frac{1}{2\eta_{i,1}}{\tilde{\theta }}_{i,1}^{T}{\tilde{\theta }}_{i,1}+\frac{1}{2\upsilon_{si}}{\tilde{\delta }}_{si}^{2}$$

where $\eta_{i,1}$ and $\upsilon_{si}>0$ are design parameters.

The derivative of (27) is obtained as follows:

(28) $$\begin{array}{cc} {\dot{V}}_{i,1}= & \;{\dot{V}}_{i,0}+z_{i,1}(z_{i,2}+\chi_{i,2}+\alpha_{i,1}+{\hat{l}}^{2}{\tilde{\varsigma }}_{i,2}+\theta_{i,1}^{T}\phi_{i,1}\left({\hat{\bar{\varsigma }}}_{i,1}\right)+{\tilde{\theta }}_{i,1}^{T}\phi \left({\hat{\bar{\varsigma }}}_{i,1}\right)\\ \; & +\varepsilon_{i,1}+\Delta \psi_{i,1}+d_{i,1}-{\dot{\bar{y}}}_{i})-\frac{1}{\eta_{i,1}}{\tilde{\theta }}_{i,1}^{T}{\dot{\theta }}_{i,1}+\frac{1}{\upsilon_{si}}{\tilde{\delta }}_{si}\left({\dot{\delta }}_{si}-{\dot{\hat{\delta }}}_{si}\right) \end{array}$$

The virtual control law $\alpha_{i,1}$, the parameter adaptation law $\theta_{i,1}$, and the sensor-fault compensation law for ${\hat{\delta }}_{si}$ are designed as follows:

(29) $$\alpha_{i,1}=-g_{i,1}{\tilde{z}}_{i,1}-\theta_{i,1}^{T}\phi_{i,1}\left({\hat{\bar{\varsigma }}}_{i,1}\right)+{\dot{\bar{y}}}_{i}$$

(30) $${\dot{\theta }}_{i,1}=\eta_{i,1}{\tilde{z}}_{i,1}\phi_{i,1}\left({\hat{\bar{\varsigma }}}_{i,1}\right)-\kappa_{i,1}\theta_{i,1}$$

(31) $${\dot{\hat{\delta }}}_{si}=-\upsilon_{si}g_{i,1}{\tilde{z}}_{i,1}-\mu_{i}{\hat{\delta }}_{si}$$

where $g_{i,1}>0$, $\kappa_{i,1}>0$ and $\mu_{i}>0$ are the parameters to be calculated.

Substituting (29)–(31) into (28) yields

(32) $$\begin{array}{cc} {\dot{V}}_{i,1}= & \;{\dot{V}}_{i,0}+z_{i,1}\left(z_{i,2}+\chi_{i,2}+{\hat{l}}^{2}{\tilde{\varsigma }}_{i,2}+{\tilde{\theta }}_{i,1}^{T}\phi_{i,1}\left({\hat{\bar{\varsigma }}}_{i,1}\right)+\varepsilon_{i,1}+\Delta \psi_{i,1}+d_{i,1}\right)\\ \; & -g_{i.1}z_{i,1}^{2}+g_{i.1}{\tilde{\delta }}_{si}^{2}-{\tilde{\theta }}_{i,1}^{T}{\tilde{z}}_{i,1}\phi_{i,1}\left({\hat{\bar{\varsigma }}}_{i,1}\right)+\frac{\kappa_{i,1}}{\eta_{i,1}}{\tilde{\theta }}_{i,1}^{T}\theta_{i,1}\\ \; & +\frac{1}{\upsilon_{si}}{\tilde{\delta }}_{si}{\dot{\delta }}_{si}+g_{i,1}{\tilde{\delta }}_{si}{\tilde{z}}_{i,1}+\frac{1}{\upsilon_{si}}{\tilde{\delta }}_{si}{\hat{\delta }}_{si} \end{array}$$

Applying Young’s inequality, we have

(33) $$\begin{array}{cc} z_{i,1}\left(z_{i,2}+\chi_{i,2}+{\hat{l}}^{2}{\tilde{\varsigma }}_{i,2}+\varepsilon_{i,1}+\Delta \psi_{i,1}+d_{i,1}\right) & \leq 3z_{i,1}^{2}+\frac{1}{2}z_{i,2}^{2}+\frac{1}{2}\chi_{i,2}^{2}+\frac{1}{2}\left({\hat{l}}^{4}+{\hat{l}}^{2}\gamma_{i,1}^{2}\right){∥{\tilde{\varsigma }}_{i}∥}^{2}+\frac{1}{2}\left(\varepsilon_{i,1}^{*2}+d_{i,1}^{*2}\right)\\ z_{i,1}{\tilde{\theta }}_{i,1}^{T}\phi_{i,1}\left({\hat{\bar{\varsigma }}}_{i,1}\right)=\left({\tilde{z}}_{i,1}-{\tilde{\delta }}_{si}\right){\tilde{\theta }}_{i,1}^{T}\phi_{i,1}\left({\hat{\bar{\varsigma }}}_{i,1}\right) & \leq {\tilde{z}}_{i,1}{\tilde{\theta }}_{i,1}^{T}\phi_{i,1}\left({\hat{\bar{\varsigma }}}_{i,1}\right)+\frac{1}{2}{\tilde{\delta }}_{si}^{2}+\frac{1}{2}{\tilde{\theta }}_{i,1}^{T}{\tilde{\theta }}_{i,1}\\ {\tilde{\theta }}_{i,1}^{T}\theta_{i,1} & \leq -\frac{1}{2}{\tilde{\theta }}_{i,1}^{T}{\tilde{\theta }}_{i,1}+\frac{1}{2}\theta_{i,1}^{*T}\theta_{i,1}^{*}\\ {\tilde{\delta }}_{si}{\dot{\delta }}_{si} & \leq \frac{1}{2}{\tilde{\delta }}_{si}^{2}+\frac{1}{2}{\bar{\bar{\delta }}}_{si}^{2}\\ {\tilde{\delta }}_{si}{\hat{\delta }}_{si} & \leq -\frac{1}{2}{\tilde{\delta }}_{si}^{2}+\frac{1}{2}{\bar{\delta }}_{si}^{2} \end{array}$$

Substituting (23) and (33) into (32) yields

(34) $$\begin{array}{cc} {\dot{V}}_{i,1}\leq & -{\bar{c}}_{i}{∥{\tilde{\varsigma }}_{i}∥}^{2}-c_{ai}{\tilde{\delta }}_{ai}^{2}-{\bar{c}}_{si}{\tilde{\delta }}_{si}^{2}-G_{i,1}z_{i,1}^{2}-\frac{1}{2}\left(\frac{\kappa_{i,1}}{\eta_{i,1}}-1-{∥P_{i}∥}^{2}{∥{\hat{L}}_{i}∥}^{2}\right){\tilde{\theta }}_{i,1}^{T}{\tilde{\theta }}_{i,1}\\ \; & +\frac{1}{2}{∥P_{i}∥}^{2}{∥{\hat{L}}_{i}∥}^{2}\sum_{m=2}^{n_{i}}{\tilde{\theta }}_{i,m}^{T}{\tilde{\theta }}_{i,m}+\frac{1}{2}\chi_{i,2}^{2}+\frac{1}{2}z_{i,2}^{2}+D_{i,1} \end{array}$$

where ${\bar{c}}_{i}=c_{i}-\frac{1}{2}{\hat{l}}^{4}-\frac{1}{2}{\hat{l}}^{2}\gamma_{i,1}^{2}$, ${\bar{c}}_{si}=\frac{\mu_{i}}{2\upsilon_{si}}-c_{si}-g_{i,1}-\frac{1}{2}-\frac{1}{2\upsilon_{si}}$, $G_{i,1}=g_{i,1}-3$, and $D_{i,1}=D_{i,0}+\frac{1}{2}\left(\varepsilon_{i,1}^{*2}+d_{i,1}^{*2}+\frac{\kappa_{i,1}}{\eta_{i,1}}{\tilde{\theta }}_{i,1}^{*T}{\tilde{\theta }}_{i,1}^{*}+\frac{\mu_{i}}{\upsilon_{si}}{\bar{\delta }}_{si}^{2}+\frac{1}{\upsilon_{si}}{\bar{\bar{\delta }}}_{si}^{2}\right)$.

Step 2: From (24), the dynamics of $z_{i,2}$ can be obtained as

(35) $$\begin{array}{c} {\dot{z}}_{i,2}=z_{i,3}+\chi_{i,3}+\alpha_{i,3}+\theta_{i,2}^{T}\phi_{i,2}\left({\hat{\bar{\varsigma }}}_{i,2}\right)+{\tilde{\theta }}_{i,2}^{T}\phi_{i,2}\left({\hat{\bar{\varsigma }}}_{i,2}\right)+\varepsilon_{i,2}+k_{2}{\hat{l}}^{2}\left(y_{i}^{f}-{\hat{\delta }}_{si}-{\hat{y}}_{i}\right)-{\dot{\zeta }}_{i,2} \end{array}$$

Choose the Lyapunov function candidate

(36) $$V_{i,2}=V_{i,1}+\frac{1}{2}z_{i,2}^{2}+\frac{1}{2\eta_{i,2}}{\tilde{\theta }}_{i,2}^{T}{\tilde{\theta }}_{i,2}+\frac{1}{2}\chi_{i,2}^{2}$$

where $\eta_{i,2}>0$ is an adaptive gain to be designed.

Differentiating (36) and substituting (35) yields

(37) $$\begin{array}{cc} {\dot{V}}_{i,2}= & \;{\dot{V}}_{i,1}+z_{i,2}(z_{i,3}+\chi_{i,3}+\alpha_{i,2}+\theta_{i,2}^{T}\phi_{i,2}\left({\hat{\bar{\varsigma }}}_{i,2}\right)+{\tilde{\theta }}_{i,2}^{T}\phi_{i,2}\left({\hat{\bar{\varsigma }}}_{i,2}\right)+\varepsilon_{i,2}\\ \; & +k_{2}{\hat{l}}^{2}\left(y_{i}^{f}-{\hat{\delta }}_{si}-{\hat{y}}_{i}\right)-{\dot{\zeta }}_{i,2})-\frac{1}{\eta_{i,2}}{\tilde{\theta }}_{i,2}^{T}{\dot{\theta }}_{i,2}+\chi_{i,2}{\dot{\chi }}_{i,2} \end{array}$$

The virtual controller $\alpha_{i,2}$ and the adaptive law $\theta_{i,2}$ are given by

(38) $$\alpha_{i,2}=-g_{i.2}z_{i,2}-\frac{3}{2}z_{i,2}-\theta_{i,2}^{T}\phi_{i,2}\left({\hat{\bar{\varsigma }}}_{i,2}\right)-k_{2}{\hat{l}}^{2}\left(y_{i}^{f}-{\hat{\delta }}_{si}-{\hat{y}}_{i}\right)+{\dot{\zeta }}_{i,2}$$

(39) $${\dot{\theta }}_{i,2}=\eta_{i,2}z_{i,2}\phi_{i,2}\left({\hat{\bar{\varsigma }}}_{i,2}\right)-\kappa_{i,2}\theta_{i,2}$$

where $g_{i,2}>0$, $\kappa_{i,2}>0$ and $\mu_{i}>0$ are design constants.

Next, by applying Young’s inequality, one has

(40) $$\begin{array}{cc} z_{i,2}\left(z_{i,3}+\chi_{i,3}+\varepsilon_{i,2}\right) & \leq \frac{3}{2}z_{i,2}^{2}+\frac{1}{2}z_{i,3}^{2}+\frac{1}{2}\chi_{i,3}^{2}+\frac{1}{2}\varepsilon_{i,2}^{*2}\\ {\tilde{\theta }}_{i,2}^{T}\theta_{i,2} & \leq -\frac{1}{2}{\tilde{\theta }}_{i,2}^{T}{\tilde{\theta }}_{i,2}+\frac{1}{2}\theta_{i,2}^{*T}\theta_{i,2}^{*} \end{array}$$

Substituting (34) and (38)–(40) into (40) yields

(41) $$\begin{array}{cc} {\dot{V}}_{i,2}\leq & -{\bar{c}}_{i}{∥{\tilde{\varsigma }}_{i}∥}^{2}-c_{ai}{\tilde{\delta }}_{ai}^{2}-{\bar{c}}_{si}{\tilde{\delta }}_{si}^{2}-\sum_{j=1}^{2}G_{i,j}z_{i,j}^{2}-\frac{1}{2}\left(\frac{\kappa_{i,1}}{\eta_{i,1}}-1-{∥P_{i}∥}^{2}{∥{\hat{L}}_{i}∥}^{2}\right){\tilde{\theta }}_{i,1}^{T}{\tilde{\theta }}_{i,1}\\ \; & +\frac{1}{2}\left(\frac{\kappa_{i,2}}{\eta_{i,2}}-{∥P_{i}∥}^{2}{∥{\hat{L}}_{i}∥}^{2}\right){\tilde{\theta }}_{i,2}^{T}{\tilde{\theta }}_{i,2}+\frac{1}{2}{∥P_{i}∥}^{2}{∥{\hat{L}}_{i}∥}^{2}\sum_{m=3}^{n_{i}}{\tilde{\theta }}_{i,m}^{T}{\tilde{\theta }}_{i,m}+\frac{1}{2}\sum_{j=1}^{2}\chi_{i,j+1}^{2}\\ \; & +\frac{1}{2}z_{i,3}^{2}-\left(\frac{\chi_{i,2}^{2}}{\tau_{i,m}}-{\dot{\alpha }}_{i,1}\chi_{i,2}\right)+D_{i,2} \end{array}$$

where $G_{i,2}=g_{i,2}-\frac{1}{2}$ and $D_{i,2}=D_{i,1}+\frac{1}{2}\varepsilon_{i,2}^{*2}+\frac{\kappa_{i,2}}{2\eta_{i,2}}{\tilde{\theta }}_{i,2}^{*T}{\tilde{\theta }}_{i,2}^{*}$.

Step *m* ($m=3,\ldots ,n_{i}-1$): The time derivative of $z_{i,m}$ is obtained as

(42) $$\begin{array}{c} {\dot{z}}_{i,m}=z_{i,m+1}+\chi_{i,m+1}+\alpha_{i,m}+\theta_{i,m}^{T}\phi_{i,m}\left({\hat{\bar{\varsigma }}}_{i,m}\right)+{\tilde{\theta }}_{i,m}^{T}\phi_{i,m}\left({\hat{\bar{\varsigma }}}_{i,m}\right)+\varepsilon_{i,m}+k_{m}{\hat{l}}^{m}\left(y_{i}^{f}-{\hat{\delta }}_{si}-{\hat{y}}_{i}\right)-{\dot{\zeta }}_{i,m} \end{array}$$

Choose the Lyapunov function candidate as follows:

(43) $$V_{i,m}=V_{i,m-1}+\frac{1}{2}z_{i,m}^{2}+\frac{1}{2\eta_{i,m}}{\tilde{\theta }}_{i,m}^{T}{\tilde{\theta }}_{i,m}+\frac{1}{2}\chi_{i,m}^{2}$$

where $\eta_{i,m}$ will be designed later.

Taking the derivative of (43) and substituting (42) gives

(44) $$\begin{array}{cc} {\dot{V}}_{i,m}= & \;{\dot{V}}_{i,m-1}+z_{i,m}(z_{i,m+1}+\chi_{i,m+1}+\alpha_{i,m}+\theta_{i,m}^{T}\phi_{i,m}\left({\hat{\bar{\varsigma }}}_{i,m}\right)+{\tilde{\theta }}_{i,m}^{T}\phi_{i,m}\left({\hat{\bar{\varsigma }}}_{i,m}\right)+\varepsilon_{i,m}\\ \; & +k_{m}{\hat{l}}^{m}\left(y_{i}^{f}-{\hat{\delta }}_{si}-{\hat{y}}_{i}\right)-{\dot{\zeta }}_{i,m})-\frac{1}{\eta_{i,m}}{\tilde{\theta }}_{i,m}^{T}{\dot{\theta }}_{i,m}+\chi_{i,m}{\dot{\chi }}_{i,m} \end{array}$$

The virtual control $\alpha_{i,m}$ and the adaptive law $\theta_{i,m}$ are designed as

(45) $$\alpha_{i,m}=-g_{i.m}z_{i,m}-\frac{5}{2}z_{i,m}-\theta_{i,m}^{T}\phi_{i,m}\left({\hat{\bar{\varsigma }}}_{i,m}\right)-k_{m}{\hat{l}}^{m}\left(y_{i}^{f}-{\hat{\delta }}_{si}-{\hat{y}}_{i}\right)+{\dot{\zeta }}_{i,m}$$

(46) $${\dot{\theta }}_{i,m}=\eta_{i,m}z_{i,m}\phi_{i,m}\left({\hat{\bar{\varsigma }}}_{i,m}\right)-\kappa_{i,m}\theta_{i,m}$$

where $g_{i,m}>0$, $\kappa_{i,m}>0$ and $\mu_{i}>0$ are the design parameters.

Using Young’s inequality, we obtain

(47) $$\begin{array}{cc} z_{i,m}\left(z_{i,m+1}+\chi_{i,m+1}+\varepsilon_{i,m}\right) & \leq \frac{3}{2}z_{i,m}^{2}+\frac{1}{2}z_{i,m+1}^{2}+\frac{1}{2}\chi_{i,m+1}^{2}+\frac{1}{2}\varepsilon_{i,m}^{2}\\ {\tilde{\theta }}_{i,m}^{T}\theta_{i,m} & \leq -\frac{1}{2}{\tilde{\theta }}_{i,m}^{T}{\tilde{\theta }}_{i,m}+\frac{1}{2}\theta_{i,m}^{*T}\theta_{i,m}^{*}. \end{array}$$

Substituting (41) and (45)–(47) into (44) yields

(48) $$\begin{array}{cc} {\dot{V}}_{i,m}\leq & -{\bar{c}}_{i}{∥{\tilde{\varsigma }}_{i}∥}^{2}-c_{ai}{\tilde{\delta }}_{ai}^{2}-{\bar{c}}_{si}{\tilde{\delta }}_{si}^{2}-\sum_{j=1}^{m}G_{i,j}z_{i,j}^{2}-\frac{1}{2}\left(\frac{\kappa_{i,1}}{\eta_{i,1}}-1-{∥P_{i}∥}^{2}{∥{\hat{L}}_{i}∥}^{2}\right){\tilde{\theta }}_{i,1}^{T}{\tilde{\theta }}_{i,1}\\ \; & +\frac{1}{2}\sum_{j=2}^{m}\left(\frac{\kappa_{i,j}}{\eta_{i,j}}-{∥P_{i}∥}^{2}{∥{\hat{L}}_{i}∥}^{2}\right){\tilde{\theta }}_{i,j}^{T}{\tilde{\theta }}_{i,j}+\frac{1}{2}{∥P_{i}∥}^{2}{∥{\hat{L}}_{i}∥}^{2}\sum_{j=m+1}^{n_{i}}{\tilde{\theta }}_{i,m}^{T}{\tilde{\theta }}_{i,m}\\ \; & +\frac{1}{2}\sum_{j=1}^{m}\chi_{i,j+1}^{2}+\frac{1}{2}z_{i,m+1}^{2}-\sum_{j=2}^{m}\left(\frac{\chi_{i,j}^{2}}{\tau_{i,j}}+{\dot{\alpha }}_{i,j-1}\chi_{i,j}\right)+D_{i,m} \end{array}$$

where $G_{i,j}=g_{i,j}-\frac{1}{2}$ and $D_{i,m}=D_{i,m-1}+\frac{1}{2}\varepsilon_{i,m}^{*2}+\frac{\kappa_{i,m}}{2\eta_{i,m}}{\tilde{\theta }}_{i,m}^{*T}{\tilde{\theta }}_{i,m}^{*}$.

Step $n_{i}$: From (24) and the observer dynamics, we have

(49) $$\begin{array}{cc} {\dot{z}}_{i,n_{i}} & ={\dot{\hat{\varsigma }}}_{i,n_{i}}-{\dot{\zeta }}_{i,n_{i}}\\ \; & =u_{i}+{\hat{\delta }}_{ai}+\theta_{i,n_{i}}^{T}\phi_{i,n_{i}}\left({\hat{\bar{\varsigma }}}_{i,n_{i}}\right)+{\tilde{\theta }}_{i,n_{i}}^{T}\phi_{i,n_{i}}\left({\hat{\bar{\varsigma }}}_{i,n_{i}}\right)+\varepsilon_{i,n_{i}}+k_{n_{i}}{\hat{l}}^{n_{i}}\left(y_{i}^{f}-{\hat{\delta }}_{si}-{\hat{y}}_{i}\right)-{\dot{\zeta }}_{i,n_{i}} \end{array}$$

The Lyapunov function for the final step is chosen as follows:

(50) $$V_{i,n_{i}}=V_{i,n_{i}-1}+\frac{1}{2}z_{i,n_{i}}^{2}+\frac{1}{2\eta_{i,n_{i}}}{\tilde{\theta }}_{i,n_{i}}^{T}{\tilde{\theta }}_{i,n_{i}}+\frac{1}{2}\chi_{i,n_{i}}^{2}$$

where $\eta_{i,n_{i}}$ will be designed later.

Differentiating (50) along (49) yields

(51) $$\begin{array}{cc} {\dot{V}}_{i,n_{i}}= & \;{\dot{V}}_{i,n_{i}-1}+z_{i,n_{i}}(u_{i}+{\hat{\delta }}_{ai}+\theta_{i,n_{i}}^{T}\phi_{i,n_{i}}\left({\hat{\bar{\varsigma }}}_{i,n_{i}}\right)+{\tilde{\theta }}_{i,n_{i}}^{T}\phi_{i,n_{i}}\left({\hat{\bar{\varsigma }}}_{i,n_{i}}\right)+\varepsilon_{i,n_{i}}\\ \; & +k_{n_{i}}{\hat{l}}^{n_{i}}\left(y_{i}^{f}-{\hat{\delta }}_{si}-{\hat{y}}_{i}\right)-{\dot{\zeta }}_{i,n_{i}})-\frac{1}{\eta_{i,n_{i}}}{\tilde{\theta }}_{i,n_{i}}^{T}{\dot{\theta }}_{i,n_{i}}+\chi_{i,n_{i}}{\dot{\chi }}_{i,n_{i}} \end{array}$$

The final control input $u_{i}$ and the adaptation law for $\theta_{i,n_{i}}$ are designed as

(52) $$u_{i}=-g_{i.n_{i}}z_{i,n_{i}}-\frac{1}{2}z_{i,n_{i}}-{\hat{\delta }}_{ai}-\theta_{i,n_{i}}^{T}\phi_{i,n_{i}}\left({\hat{\bar{\varsigma }}}_{i,n_{i}}\right)-k_{n_{i}}{\hat{l}}^{n_{i}}\left(y_{i}^{f}-{\hat{\delta }}_{si}-{\hat{y}}_{i}\right)+{\dot{\zeta }}_{i,n_{i}}$$

(53) $${\dot{\theta }}_{i,n_{i}}=\eta_{i,n_{i}}z_{i,n_{i}}\phi_{i,n_{i}}\left({\hat{\bar{x}}}_{i,n_{i}}\right)-\kappa_{i,n_{i}}\theta_{i,n_{i}}$$

where $\eta_{i,n_{i}}$ will be designed later.

Using Young’s inequality, we obtain

(54) $$\begin{array}{cc} z_{i,n_{i}}\varepsilon_{i,m} & \leq \frac{1}{2}z_{i,n_{i}}^{2}+\frac{1}{2}\varepsilon_{i,m}^{2}\\ {\tilde{\theta }}_{i,n_{i}}^{T}\theta_{i,n_{i}} & \leq -\frac{1}{2}{\tilde{\theta }}_{i,n_{i}}^{T}{\tilde{\theta }}_{i,n_{i}}+\frac{1}{2}\theta_{i,n_{i}}^{*T}\theta_{i,n_{i}}^{*}\\ {\dot{\alpha }}_{i,j-1}\chi_{i,j} & \leq \frac{1}{2}{\dot{\alpha }}_{i,j-1}^{2}\chi_{i,j}^{2}+\frac{1}{2} \end{array}$$

There is a constant ${\bar{\bar{\alpha }}}_{i,j-1}$ satisfying $\left|{\dot{\alpha }}_{i,j-1}\right|\leq {\bar{\bar{\alpha }}}_{i,j-1}$ is found. Substituting (48) and (52)–(54) into (51) yields

(55) $$\begin{array}{cc} {\dot{V}}_{i,n_{i}}\leq & -{\bar{c}}_{i}{∥{\tilde{\bar{\varsigma }}}_{i}∥}^{2}-c_{ai}{\tilde{\delta }}_{ai}^{2}-{\bar{c}}_{si}{\tilde{\delta }}_{si}^{2}-\sum_{j=1}^{n_{i}}G_{i,j}z_{i,j}^{2}-\frac{1}{2}\left(\frac{\kappa_{i,1}}{\eta_{i,1}}-1-{∥P_{i}∥}^{2}{∥{\hat{L}}_{i}∥}^{2}\right){\tilde{\theta }}_{i,1}^{T}{\tilde{\theta }}_{i,1}\\ \; & +\frac{1}{2}\sum_{j=2}^{n_{i}}\left(\frac{\kappa_{i,j}}{\eta_{i,j}}-{∥P_{i}∥}^{2}{∥{\hat{L}}_{i}∥}^{2}\right){\tilde{\theta }}_{i,j}^{T}{\tilde{\theta }}_{i,j}-\frac{1}{2}\sum_{j=2}^{n_{i}}\left(\frac{2}{\tau_{i,j}}-{\bar{\bar{\alpha }}}_{i,j-1}-1\right)\chi_{i,j}^{2}+D_{i,n_{i}} \end{array}$$

where $D_{i,n_{i}}=D_{i,n_{i}-1}+\frac{1}{2}\varepsilon_{i,n_{i}}^{*2}+\frac{\kappa_{i,n_{i}}}{2\eta_{i,n_{i}}}{\tilde{\theta }}_{i,n_{i}}^{*T}{\tilde{\theta }}_{i,n_{i}}^{*}+\frac{n-1}{2}$.

Simplifying, (55) can be rewritten as follows:

(56) $${\dot{V}}_{i,n_{i}}\leq -C_{i}V_{i,n_{i}}+D_{i,n_{i}},\left(i=1,\ldots ,N\right)$$

where $C_{i}=\min\left\{\begin{array}{c} \frac{{\bar{c}}_{i}}{\lambda_{\max}{\left(P\right)}_{i}},2c_{ai}\upsilon_{ai},2c_{si}\upsilon_{si},2G_{i,n_{i}},\left(\frac{\kappa_{i,1}}{\eta_{i,1}}-1-{∥P_{i}∥}^{2}{∥{\hat{L}}_{i}∥}^{2}\right),\;\\ \left(\frac{\kappa_{i,j}}{\eta_{i,j}}-{∥P_{i}∥}^{2}{∥{\hat{L}}_{i}∥}^{2}\right),\left(\frac{2}{\tau_{i,j}}-{\bar{\bar{\alpha }}}_{i,j-1}-1\right) \end{array}\right\}>0$.

**Theorem** **2.** *For the nonlinear MASs (4) with actuator and sensor faults (2), by designing the distributed output predictor (6), the state and actuator fault observer (17), the virtual controllers (29), (38), (45), the sensor fault estimation adaptive law (31), the final controller (52), and the parameters adaptive laws (30), (39), (46), and (53), all the signals of the MASs are bounded, and the consensus tracking error converges to a small neighborhood of the origin.*

**Proof.**  Consider the composite Lyapunov function

(57) $$V=\sum_{i=1}^{N}V_{i,n_{i}}$$

From (56), it follows that

(58) $$\dot{V}\leq -CV+D$$

where $C=\min\{C_{i},\;i=1,2,\ldots ,N\}$ and $D=\sum_{i=1}^{N}D_{i,n_{i}}$. Solving the differential inequality (58) yields

(59) $$0\leq {∥z_{i,1}∥}^{2}\leq V\left(t\right)\leq e^{-Ct}V\left(0\right)+\frac{D}{C}\left(1-e^{-Ct}\right)$$

Apparently, inequality (59) shows that the output $y_{i}$ can be controlled to track the virtual signal ${\bar{y}}_{i}$ of the output predictor with a small tracking error by selecting suitable parameters. The output of all followers in graph G tracks to the leader’s trajectory with bounded residual errors, and all the variables of the closed-loop MAS are bounded. The proof is completed. □

**Remark** **6.** *Inequality (59) indicates that the performance of the MAS is influenced by the design parameters through C and D. In particular, increasing C and decreasing D improves the convergence speed and steady-state accuracy. However, the parameters $\mu_{i}$, $\upsilon_{si}$, $\kappa_{i,m}$, and $\eta_{i,m}$ affect $C_{i}$ and $D_{i,n_{i}}$ simultaneously. For example, increasing $\mu_{i}$ or $\kappa_{i,m}$ and decreasing $\upsilon_{si}$ or $\eta_{i,m}$ may enlarge $C_{i}$ but also increase $D_{i,n_{i}}$, leading to a trade-off between transient response and steady-state performance. In practical applications, these parameters should be tuned according to the desired convergence rate and allowable steady-state error of the MASs.*

**Remark** **7.** *The proposed hierarchical FTC framework improves scalability and reduces design complexity by avoiding explicit fault detection and isolation. In traditional FDI-based FTC schemes, fault diagnosis, isolation, and controller reconfiguration are often designed as separate modules, which increases system complexity and requires additional logic for fault classification and switching. In contrast, the proposed framework integrates fault estimation and compensation into a unified adaptive structure, allowing the controller to accommodate faults online without requiring fault identification or mode switching. This hierarchical and fully distributed design enables a straightforward extension to large-scale multi-agent systems with minimal redesign effort.*

## 4. Simulation Results

**Figure 1:**
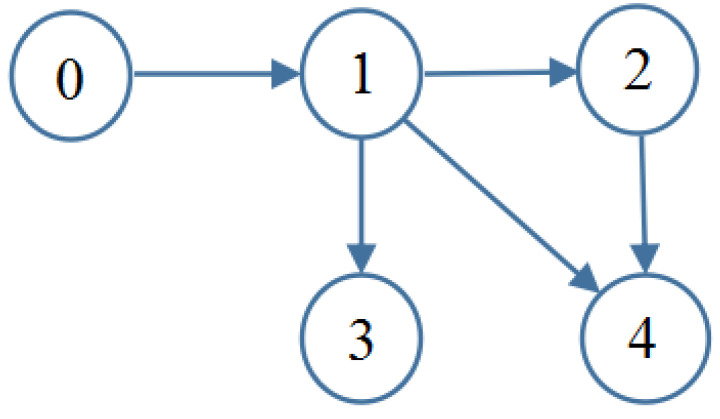
Directed algebraic topology.

In this section, the MASs consisting of one leader (indexed by 0) and four followers (indexed by 1–4) are considered, as shown in Figure 1. Two simulation examples are provided to verify the feasibility and superiority of the aforementioned control strategy.

Example 1: Consider the heterogeneous follower dynamics given by

(60) $$\left\{\begin{array}{c} {\dot{\varsigma }}_{i,1}=\varsigma_{i,2}+\psi_{i,1}\left(\varsigma_{i,1}\right)+d_{i,1}\\ {\dot{\varsigma }}_{i,2}=\beta_{ai}\left(t\right)u_{i}+f_{ai}\left(t\right)+\psi_{i,2}\left({\bar{\varsigma }}_{i,2}\right)+d_{i,2}\\ y_{i}^{f}=\beta_{si}\left(t\right)\varsigma_{i,1}+f_{si}\left(t\right),i=1,2,\ldots ,4 \end{array}\right.$$

where the nonlinear functions are selected as $\psi_{1,1}\left(\varsigma_{1,1}\right)=0$, $\psi_{1,2}\left({\bar{\varsigma }}_{1,2}\right)=2\sin\left(\varsigma_{1,1}\varsigma_{1,2}\right)$, $\psi_{2,1}\left(\varsigma_{2,1}\right)=\varsigma_{2,1}\cos\left(\varsigma_{2,1}\right)$, $\psi_{2,2}\left({\bar{\varsigma }}_{2,2}\right)=2\sin\left(\varsigma_{2,1}\varsigma_{2,2}\right)+\varsigma_{2,2}$, $\psi_{3,1}\left(\varsigma_{3,1}\right)=\cos\left(\varsigma_{3,1}\right)e^{-\varsigma_{3,1}^{2}}$, $\psi_{3,2}\left({\bar{\varsigma }}_{3,2}\right)=\sin\left(\varsigma_{3,1}\varsigma_{3,2}\right)e^{-\varsigma_{3,1}^{2}}$, and $\psi_{4,1}\left(\varsigma_{4,1}\right)=\sin\left(\varsigma_{4,1}\right)e^{-\varsigma_{4,1}^{2}}$, $\psi_{4,2}\left({\bar{\varsigma }}_{4,2}\right)=\varsigma_{4,1}\sin\left(\varsigma_{4,1}\varsigma_{4,2}\right)+\varsigma_{4,2}^{2}$. The external disturbances are chosen as $d_{i,1}=0.1\sin\left(t\right)$ and $d_{i,2}=0.2\cos\left(t\right)$. The trajectory of leader is expressed as $y_{d}=\sin\left(t\right)$. The actuator and sensor fault parameters are selected as $\beta_{a1}=\beta_{a2}=\beta_{a3}=\beta_{s1}=\beta_{s4}=0.8$, $\beta_{a4}=\beta_{s2}=\beta_{s3}=0.2\sin\left(t\right)+0.8$, $f_{a1}=f_{a2}=f_{s1}=0.2$, $f_{a4}=f_{s2}=0$, and $f_{a3}=f_{s3}=f_{s4}=0.2\sin\left(t\right)$. The fuzzy basis functions are defined as $\mu_{F_{i}^{l}}\left({\hat{\varsigma }}_{i,m}\right)=\exp(-\left({\left({\hat{\varsigma }}_{i,m}+\frac{\pi }{3}-\left(l-1\right)\frac{\pi }{6}/\frac{\pi }{12}\right)}^{2}\right)$, l=1,2,…,5.

The design parameters are selected as l=5, $k_{1}=8$, $k_{2}=20$, $k_{ai}=0.5$, $\upsilon_{ai}=2$, $\sigma_{ai}=5$, $g_{i,1}=10$, $g_{i,2}=9$, $\eta_{i,1}=\eta_{i,2}=0.1$, $\mu_{si}=25$, $\kappa_{i,1}=\kappa_{i,2}=0.4$, and $\tau_{i,2}=0.01\left(i=1,\ldots ,4\right)$. The initial conditions of the follower states are $\varsigma_{1}\left(0\right)={\left[0.4,\;0.5\right]}^{T}$, $\varsigma_{2}\left(0\right)={\left[0.2,\;0.3\right]}^{T}$, $\varsigma_{3}\left(0\right)={\left[1,\;0.5\right]}^{T}$, and $\varsigma_{4}\left(0\right)={\left[0.3,\;0.4\right]}^{T})$. The initial predictor and observer states are chosen as $\bar{y}\left(0\right)={\left[0.5,\;0.6,\;0.7,\;0.8\right]}^{T}$ and ${\hat{\varsigma }}_{i}\left(0\right)={\left[0.3,\;0.5\right]}^{T}\left(i=1,\ldots ,4\right)$, respectively. The initial parameter estimates and fault estimates are $\theta_{i,m}\left(0\right)=0$ and ${\hat{\delta }}_{ai}\left(0\right)={\hat{\delta }}_{si}\left(0\right)=0\left(i=1,\ldots ,4\right)$.

**Figure 2:**
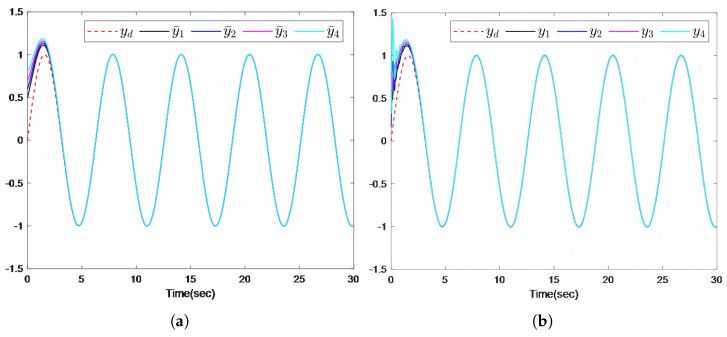
(**a**) The output trajectories of the leader and the predictors. (**b**) The output trajectories of the leader and the followers.

**Figure 3:**
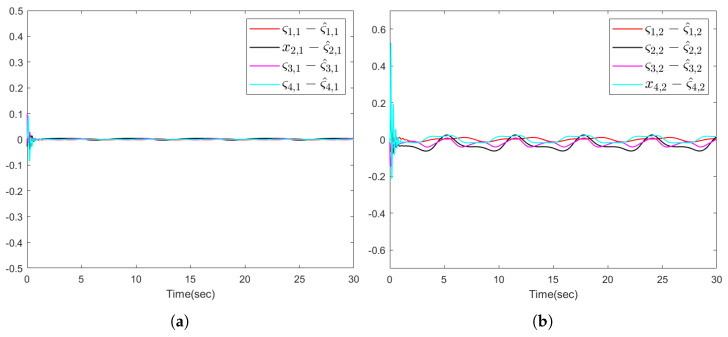
(**a**) The observer error $\varsigma_{i,1}-{\hat{\varsigma }}_{i,1}$ of all followers. (**b**) The observer error $\varsigma_{i,2}-{\hat{\varsigma }}_{i,2}$ of all followers.

**Figure 4:**
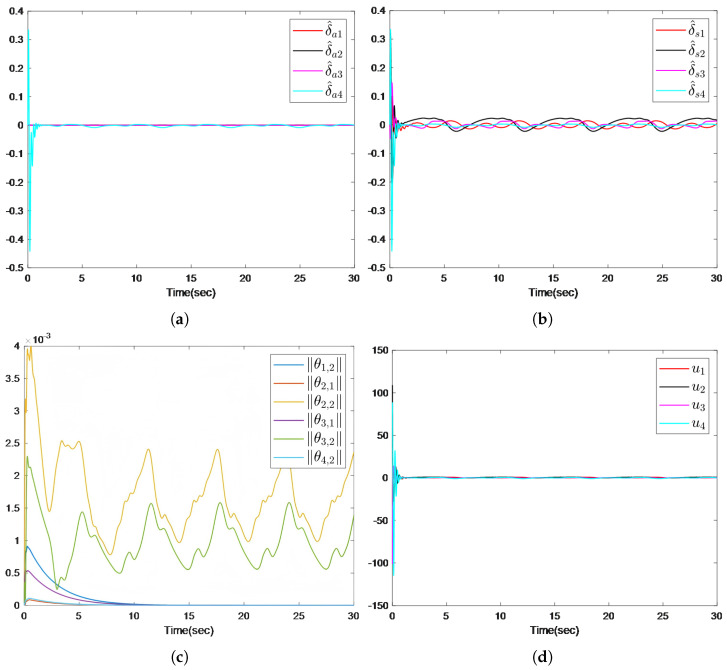
(**a**) Trajectories ${\hat{\delta }}_{ai}$ of actuator fault. (**b**) Trajectories ${\hat{\delta }}_{si}$ of sensor fault. (**c**) The trajectories of parameters adaptive law $∥\theta_{i,j}∥$. (**d**) The trajectories of control input $u_{i}$.

The simulation results are presented in Figure 2, Figure 3 and Figure 4. Figure 2a illustrates that the distributed predictor outputs ${\bar{y}}_{i}$ successfully track the leader trajectory $y_{d}$. Figure 2b further shows that the outputs of all follower agents converge to the leader trajectory. As depicted in Figure 3, the observer estimation errors converge to a small neighborhood of zero. The actuator and sensor fault estimations used for compensating the unknown faults are shown in Figure 4a,b. Figure 4c,d demonstrates that the control inputs $u_{i}$(i=1,…,4) and the adaptive parameter norms $|\theta_{i,j}|$(i=1,…,4;,j=1,2) remain bounded throughout the operation.

**Figure 5:**
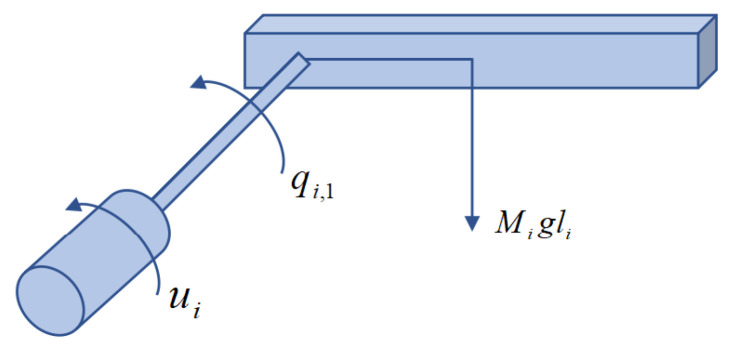
Single-link robot arm.

Example 2: In practical engineering applications, Lagrangian dynamic models are widely used for industrial system modeling, including vehicle motion systems, inverted pendulum systems, and robotic manipulators. In this example, we adopt the second-order Lagrangian dynamics presented in [40]. The communication topology of the multi-agent system remains identical to that shown in Figure 1. The leader’s trajectory is selected as $y_{d}=\sin\left(0.5t\right)+\sin\left(1.5t\right)$. Four single-link robotic arms are considered as follower agents. Each robotic arm consists of a rigid link driven by a DC motor through a gear transmission mechanism, as illustrated in Figure 5. Unlike the scenario studied in [40], the follower agents here are subject to external disturbances as well as time-varying actuator and sensor faults. The dynamics of the followers are described as

(61) $$\left\{\begin{array}{c} {\dot{q}}_{i,1}=q_{i,2}+d_{i,1}\\ {\dot{q}}_{i,2}=J_{i}^{-1}\left(\beta_{ai}\left(t\right)u_{i}+f_{ai}\left(t\right)+B_{i}q_{i,2}-M_{i}gl_{i}\sin\left(q_{i,1}\right)+d_{i,2}\right)\\ y_{i}^{f}=\beta_{si}\left(t\right)q_{i,1}+f_{si}\left(t\right),i=1,2,\cdots ,4 \end{array}\right.$$

where $q_{i,1}$ and $q_{i,2}$ denote the angle and angular velocity of the link, respectively. $J_{i}$ is the inertia of the link and motor, $B_{i}$ is the overall damping coefficient, $M_{i}$ is the link total mass, *g* is the gravity coefficient, and $l_{i}$ is the location of the link center of mass. The external disturbances are given by $d_{i,1}=0.1\sin\left(t\right)$ and $d_{i,2}=0.2\cos\left(t\right)$. The time-varying actuator and sensor faults are modeled as $\beta_{ai}\left(t\right)=0.5$, $f_{ai}\left(t\right)=0.5\cos\left(t\right)$, $\beta_{si}\left(t\right)=0.8$ and $f_{si}\left(t\right)=0.5\sin\left(t\right),i=1,2,\ldots ,4$.

For the simulation, the parameters of the followers are given as ${\left[J_{1},J_{2},J_{3},J_{4}\right]}^{T}={\left[6.9667,7.7,8.46,10.2\right]}^{T}$, $B_{i}=30.5,i=1,2,\ldots ,4$, g=9.8 and ${\left[l_{1},l_{2},l_{3},l_{4}\right]}^{T}={\left[0.6,0.8,1,1.2\right]}^{T}$. In accordance with Theorem 1, the design parameters are chosen as l=30, $k_{1}=5$, $k_{2}=10$, $k_{ai}=2$, $\upsilon_{ai}=2$, $\sigma_{ai}=10$, $g_{i,1}=16$, $g_{i,2}=10$, $\eta_{i,1}=\eta_{i,2}=0.1$, $\mu_{si}=25$, $\kappa_{i,1}=\kappa_{i,2}=0.4$, and $\tau_{i,2}=0.01\left(i=1,\ldots ,4\right)$. The initial states of the followers are set as $q_{1}\left(0\right)={\left[1,\;0.5\right]}^{T}$, $q_{2}\left(0\right)={\left[0.5,\;0.2\right]}^{T}$, $q_{3}\left(0\right)={\left[0.6,\;0\right]}^{T}$, and $q_{4}\left(0\right)={\left[1,\;0\right]}^{T}$. The initial predictor state is $\bar{y}\left(0\right)={\left[0.5,\;0.6,\;0.7,\;0.8\right]}^{T}$. The fuzzy observer initial conditions are ${\hat{q}}_{i}\left(0\right)={\left[0.3,\;0.5\right]}^{T}\left(i=1,\ldots ,4\right)$. The parameter vectors are initialized as $\theta_{i,m}\left(0\right)=0$. The initial estimates of actuator and sensor fault parameters are ${\hat{\delta }}_{ai}\left(0\right)={\hat{\delta }}_{si}\left(0\right)=0\left(i=1,\ldots ,4\right)$.

**Figure 6:**
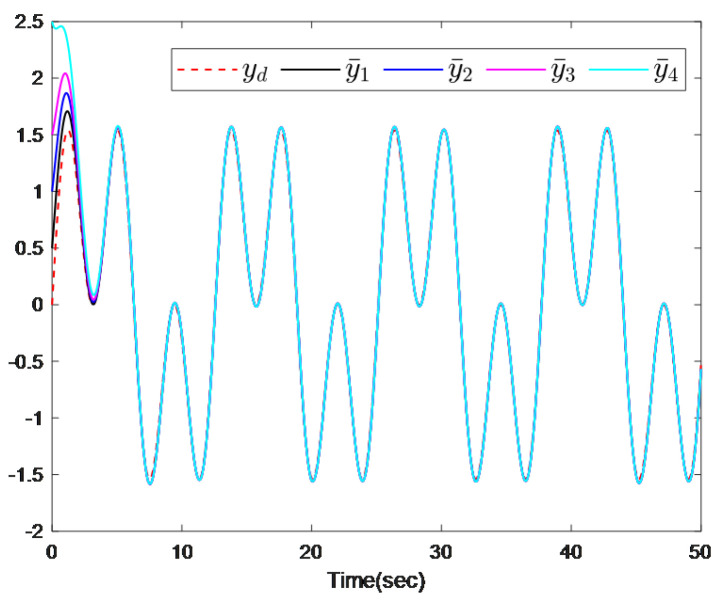
The output trajectories of leader and predictors.

**Figure 7:**
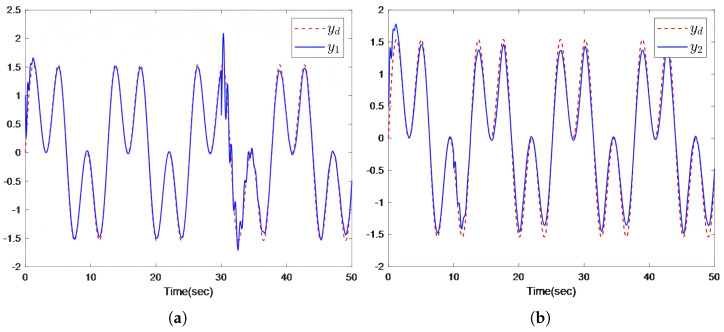

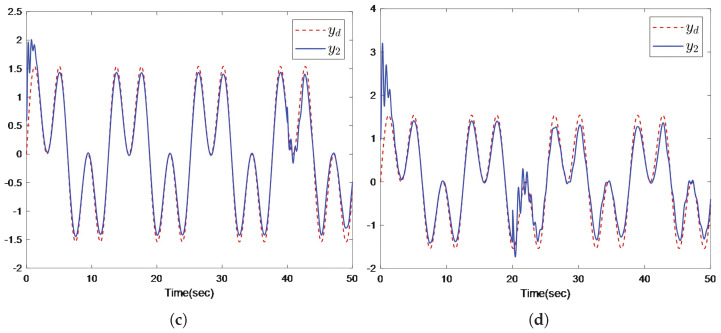
(**a**) Output trajectories of the leader and follower 1 when faults occur at t=30s. (**b**) Output trajectories of the leader and follower 2 when faults occur at t=10s. (**c**) Output trajectories of the leader and follower 3 when faults occur at t=40s. (**d**) Output trajectories of the leader and follower 4 when faults occur at t=20s.

The simulation results are presented in Figure 6 and Figure 7. Figure 6 demonstrates that all output predictors synchronize with the leader’s trajectory. Figure 7 further shows that, despite the presence of actuator and sensor faults occurring at different times, the outputs of all followers successfully track the leader’s trajectory.

## 5. Conclusions

This study developed a hierarchical fuzzy adaptive fault-tolerant consensus tracking framework for high-order nonlinear MASs subject to external disturbances and time-varying actuator and sensor faults. A differentiator-based distributed output predictor was first introduced to generate fault-insulated auxiliary signals and prevent fault propagation across the network. A fuzzy adaptive observer was then constructed to simultaneously reconstruct unmeasured states and actuator faults. Building on these estimation modules, an adaptive fault-tolerant controller together with a sensor-fault compensation mechanism was designed via the backstepping method and Lyapunov analysis. The resulting scheme ensures that all followers reliably track the leader’s trajectory while maintaining bounded closed-loop signals and effectively mitigating both actuator and sensor faults. Simulation studies validate the robustness and performance of the proposed control strategy. Overall, the framework provides a unified and practical solution for fault-tolerant cooperative control of nonlinear MASs, and future efforts will extend the approach to scenarios involving communication-link failures and hardware-level degradations.

## Data Availability

All relevant data are contained within this paper.
